# Tregitopes regulate the tolerogenic immune response and decrease the foetal death rate in abortion-prone mouse matings

**DOI:** 10.1038/s41598-020-66957-z

**Published:** 2020-06-29

**Authors:** Anna Ewa Kedzierska, Daria Lorek, Anna Slawek, Anna Chelmonska-Soyta

**Affiliations:** 1 Hirszfeld Institute of Immunology and Experimental Therapy, Polish Academy of Sciences, Wroclaw, Poland; 2 Łukasiewicz Research Network – PORT Polish Center for Technology Development, Stablowicka 147 Str., Wroclaw, Poland; 3 Wroclaw University of Environmental and Life Sciences, Wroclaw, Poland

**Keywords:** Reproductive biology, Immunology

## Abstract

The imbalance in immune tolerance may cause the variety of reproductive failures. An intravenous immunoglobulin infusion (IVIg) therapy is used to improve the live birth rate in women suffering from recurrent pregnancy loss, recurrent spontaneous abortions and recurrent implantation failures. However, the results of IVIg studies are still inconclusive as IVIg infusion in women suffering from pregnancy loss is sometimes ineffective. One of the mechanisms of action of this treatment is inhibition of B cells differentiation and expansion of Tregs and secretion of interleukin 10. It was proposed that immunomodulatory effects of IVIg may be attributed to tregitopes - self-IgG-derived epitopes present in the structure of immunoglobulins. Similarly to IVIg, tregitopes cause the expansion of Tregs and secretion of antigen-specific effector cytokine response. Here, we studied whether the administration of mouse tregitope 167 and/or 289 can prevent abortions in mouse abortion-prone mouse matings. We revealed that tregitopes reduce the foetal death rate. This may be driven by observed higher pool of peripheral Tregs, increased production of IL-10 by Tregs and Bregs and/or maintaining the tolerogenic phenotype of antigen-presenting cells. We believe that our findings may indicate a potential alternative to IVIg for therapeutic intervention in case of pregnancy failures.

## Introduction

The presence of an antigenically foreign foetus induces a state of immune tolerance in the mother organism that is crucial to embryo implantation and foetus development. Imbalances in immune tolerance may cause a variety of reproductive failures such as preeclampsia or spontaneous and recurrent miscarriage. It is estimated that the problem of miscarriage affects one in four recognized pregnancies, with 85% of them being lost in the first trimester^1,2^. Despite continuous advances in reproductive medicine, the problem of recurrent miscarriage is still unsolved, and it is believed that more than half of such pregnancies do not have a clearly defined aetiology^3^. Therapies that have been proposed for the treatment of autoimmune-mediated pregnancy/reproductive failure^4^ include intravenous infusion of immunoglobulin (IVIg), which contains a broad range of antibodies derived from the pooled plasma of healthy donors. In past years, IVIg has been used to improve the live birth rate in women suffering from recurrent pregnancy loss (RPL)^5–7^, recurrent spontaneous abortion (RSA)^8–10^ and recurrent implantation failure (RIF)^11–14^. The major mechanism of action of IVIg is related to natural killer cell inhibition, modulation of the functions of antigen-presenting cells (APCs), neutralization of cytokines and autoantibodies, inhibition of B cell differentiation and expansion of regulatory T lymphocytes (Tregs)^15–20^. A hypothesis involving Tregs expansion and secretion of tolerogenic interleukin 10 (IL-10) after IVIg treatment has been described by de Groot and co-workers^21^. They proposed that nonspecific antibodies present in IVIg might be internalized and processed by APCs and that self-immunoglobulin-derived epitopes are then presented to Tregs via the major histocompatibility complex II (MHCII). Formation of the TCR (T-cell receptor) epitope-MHCII complex leads to a decrease in the production of costimulatory molecules (CD80, CD86) by APCs and activation and proliferation of Tregs. Therefore, these natural Treg epitopes, with high-affinity binding to human class II Major Histocompatibility Complexes (HLA class-II) that are responsible for the suppression of immune response’s effector phase towards own antigens were called tregitopes (T regulatory cell epitopes)^21^. Many recent studies have shown that T cells exhibit a typical CD4^+^CD25^+^FOXP3^+^ regulatory cell phenotype in response to mouse and human tregitopes. *In vitro* co-incubation of T cells with immunogenic peptides inhibits the effector T cell response and the initiation of an antigen-specific effector cytokine response^22^. The sequences of natural Treg epitopes are highly conserved and are found in the light and heavy chains of human and mouse immunoglobulins (IgG). Two of five identified tregitopes, tregitope 167 (located in the first constant domain C_H_1) and tregitope 289 (located in the second constant domain C_H_2), bind to HLA class-II with the highest affinity as calculated by EpiMatrix scores^21^. Tregitopes have already been shown to regulate the immune response by increasing the expansion of Tregs in several autoimmune diseases, e.g., mouse models of diabetes^23^, experimental autoimmune encephalomyelitis (EAE)^24^ and cockroach allergy^25^. In mammalian pregnancy, Tregs are essential for the development of tolerance to foetal antigens. In both humans and mice, the levels and the activity of Tregs increase during normal pregnancy compared to non-pregnant controls and decrease in cases of spontaneous abortion when compared to normal pregnancy but not to non-pregnant subjects^26–31^. It was also recently shown that regulatory B lymphocytes (Bregs) may contribute to pregnancy maintenance based on the fact that, their number increases during normal pregnancy when compared to non-pregnant subjects and decreases in abortion-prone mice after mating and in spontaneous abortions cases in comparison to healthy pregnant women^32–35^. In CBA/JxDBA/2J mice, the most widely studied animal model of pregnancy failure caused by immune imbalance, the occurrence of abortion can be reduced by the adoptive transfer of regulatory B and/or T cells^36,37^ and, most importantly, by IVIg administration^38,39^. In this mating, the high abortion rate may be provoked by an adverse reaction against paternal antigens present in the semen of DBA/2 J males^40^. This semen induce unfavourable immune response disrupting pregnancy tolerance what may lead to increased abortion rate in mated CBA/J females. As previously mentioned, it was already shown that tregitopes are able to induce expansion of Tregs and effectively suppress adverse immune response caused by auto- and alloantigens. Moreover, simultaneous co-administration of autoantigen and tregitopes to non-obese diabetic mice induced antigen-specific adaptive tolerance more effectively than tregitopes alone^41^. Therefore, in our study we propose that semen antigens together with early (within eight hours after mating) administration of tregitopes may suppress effector immune response against delivered antigens. Thus, the aim of this study was to investigate whether the early administration of two selected IgG-derived epitopes, mouse tregitopes 167 and 289 can cause the expansion of regulatory lymphocytes and prevent abortion in a mouse abortion-prone model.

## Results

### Tregitopes decrease the abortion rate

**Figure 1:**
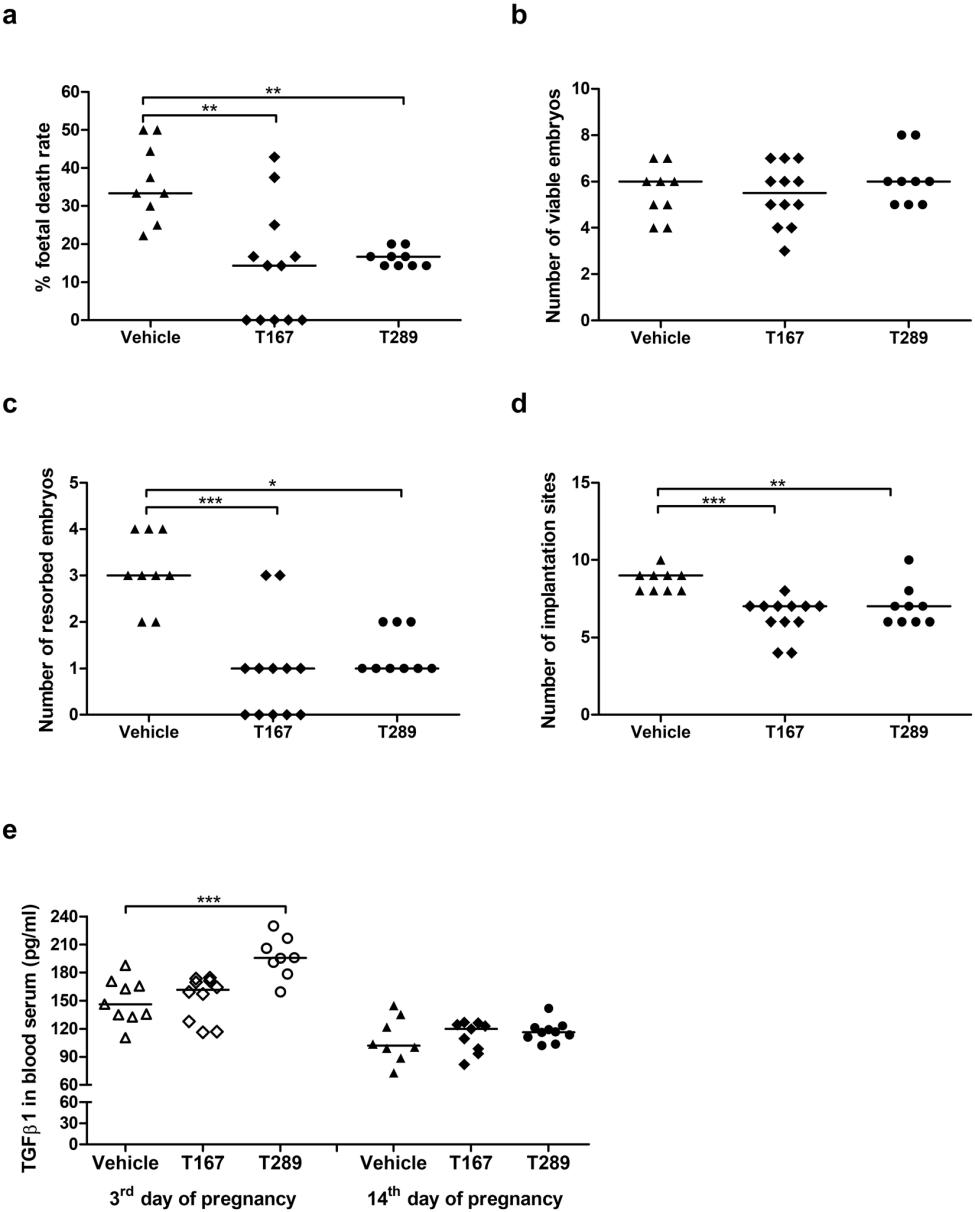
Effect of tregitope treatment on foetal death rates, numbers of viable and resorbed embryos, total implantation sites and cytokine levels in a murine abortion-prone pregnancy model. **(a)** Effect of tregitope injection on foetal death rate and numbers of **(b)** viable, **(c)** resorbed embryos and **(d)** total implantation sites evaluated at the 14^th^ day of pregnancy. **(e)** Effect of tregitope injection on TGFβ1 concentrations in serum on the 3^rd^ and 14^th^ days of pregnancy. The data were analysed by the Kruskal-Wallis test (non-normal distribution) with Dunn’s multiple comparison post-hoc test (P < 0.05) and are presented as individual values with median (at 14dpc n = 12 for T167, n = 9 for T289, n = 9 for Vehicle; at 3dpc n = 11 for T167, n = 11 for T289, n = 9 for Vehicle); *P < 0.05, **P < 0.01, and ***P < 0.001.

To determine whether tregitope administration is beneficial to pregnancy maintenance in abortion-prone mice, we calculated the foetal death rate at the 14^th^ day of pregnancy according to the formula described in the Methods section. Administration of tregitope 167 (T167) or tregitope 289 (T289) resulted in a significant decrease (p = 0.0009 and p = 0.0059, respectively) in the foetal death rate compared to the foetal death rate in female mice that received only PBS (14.29% and 16.35%, respectively, vs 36.20%) (Fig. 1a). The injection of tregitopes did not change the number of viable embryos (Fig. 1b), however, administration of either of the tested tregitopes led to a significant reduction in the number of resorbed embryos (p = 0.0001 for T167 and p = 0.0112 for T289) and total implantation sites (p = 0.0004 for T167 and p = 0.0082 for T289) compared to the control (Fig. 1c,d respectively).

### Tregitope 289 enhances the TGFβ1 level

To determine whether tregitope treatment restores the Th1/Th2 balance during pregnancy in abortion-prone mice, we examined the levels of IL-2, IL-4, IL-10, IFNγ and TGFβ1 cytokines in blood sera. The absorbances obtained for IL-2, IL-4, IL-10 and IFNγ on 3^rd^ and 14^th^ day of pregnancy were below the level of detection provided by the kit (data not shown). However, on the 3^rd^ day of pregnancy, mice treated with tregitope 289 had higher serum concentrations of TGFβ1 (196.6 ± 21.85) than control mice (149.8 ± 22.25) (Fig. 1d; p = 0.0003). At the 14^th^ day of pregnancy, we did not observe any differences in the TGFβ1 levels in the investigated groups of mice.

### Tregitopes administration increased Tregs frequency and IL-10 production

**Figure 2:**
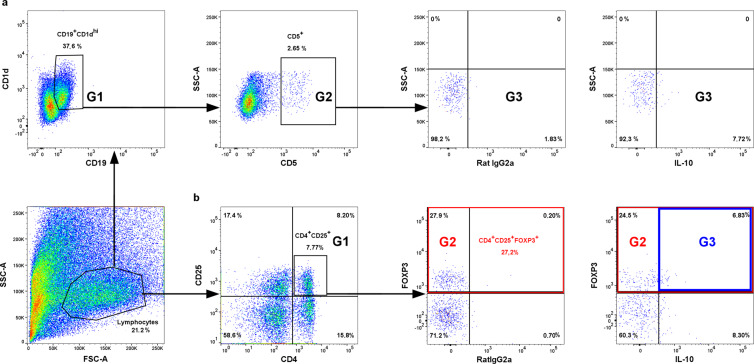
Representative dot plots for the process of regulatory T and B lymphocytes gating in spleen sample. **(a)** Gating strategy for CD19^+^CD1d^+^CD5^+^IL-10^+^ lymphocytes: **G1** represents CD19^+^CD1d^+^ cells, **G2** represents CD19^+^CD1d^+^CD5^+^ cells, and **G3** represents CD19^+^CD1d^+^CD5^+^IL-10^+^ cells. **(b)** Gating strategy for CD4^+^CD25^+^FOXP3^+^IL-10^+^ lymphocytes: **G1** represents CD4^+^CD25^+^ cells, **G2** represents overall CD4^+^CD25^+^FOXP3^+^ cells, and **G3** represents CD4^+^CD25^+^FOXP3^+^IL-10^+^ cells.

**Figure 3:**
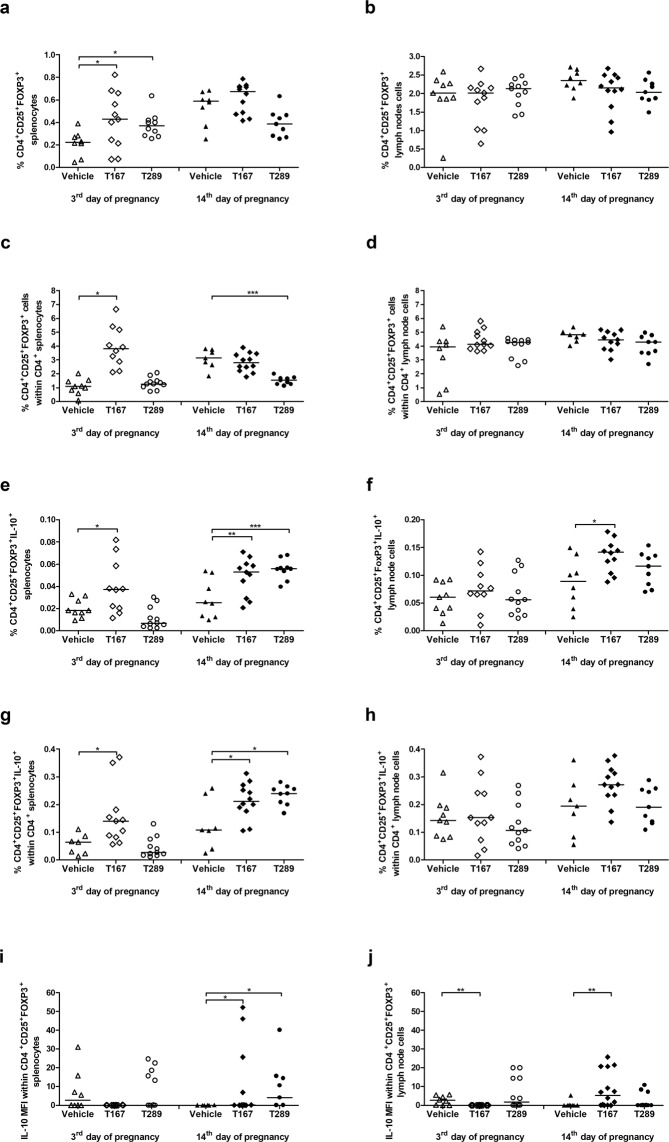
Effect of tregitope treatment on regulatory T lymphocytes in a murine abortion-prone pregnancy model. Cells were stimulated with PMA and ionomycin in the presence of brefeldin A and monensin and the frequencies of CD4^+^CD25^+^Foxp3^+^ **(a)** splenocytes and **(b)** uterine-draining lymph node cells within CD4^+^ **(c)** splenocytes and **(d)** lymph node cells and the frequencies of CD4^+^CD25^+^FOXP3^+^ IL-10^+^ splenocytes **(e)** and **(f)** lymph node cells within CD4^+^ **(g)** splenocytes and **(h)** lymph nodes and the median fluorescence intensity (MFI) of IL-10 within CD4^+^CD25^+^FOXP3^+^ **(i)** splenocytes and **(j)** uterine-draining lymph node cells were measured. The data were analysed by one-way ANOVA (normal distribution) or the Kruskal-Wallis test (non-normal distribution) with Dunn’s multiple comparison post hoc test (P < 0.05) and are presented as individual values with median (at 14dpc n = 12 for T167, n = 9 for T289, n = 9 for Vehicle; at 3dpc n = 11 for T167, n = 11 for T289, n = 9 for Vehicle). *P < 0.05, **P < 0.01, and ***P < 0.001.

To determine whether tregitopes administration to abortion-prone mice after mating could stimulate Tregs expansion, the percentage of CD4^+^CD25^+^FOXP3^+^ and CD4^+^CD25^+^FOXP3^+^IL-10^+^ cells in the spleens and uterine-draining lymph nodes (Fig. 3) of the animals were analysed based on the gating strategy shown in Fig. 2.

We found that at the 3^rd^ day of pregnancy, both tregitopes induced expansion of CD4^+^CD25^+^FOXP3^+^ cell populations in the spleens of the treated animals (T167 p = 0.0245; T289 p = 0.0399) (Fig. 3a) but not in the uterine-draining lymph nodes (Fig. 3b). The frequency of CD4^+^CD25^+^FOXP3^+^ cells in the spleen was almost two-fold higher in the treated animals (0.4212 ± 0.2498 for T167 and 0.3764 ± 0.1113 for T289) than in the vehicle group (0.2139 ± 0.1120). However, only T167 induced a higher percentage of CD4^+^CD25^+^FOXP3^+^ cells in the CD4^+^ splenocyte population (Fig. 3c; p = 0.041). We did not observe any alterations in the frequencies of CD4^+^CD25^+^FOXP3^+^ cells among CD4^+^ lymph node cells (Fig. 3d). Analysis of CD4^+^CD25^+^FOXP3^+^IL-10^+^ cells on the 3^rd^ day of pregnancy revealed that only T167 administration significantly increased the frequency of Tregs producing IL-10 in splenocytes (Fig. 3e; p = 0.0249) and in splenic CD4^+^ cells (Fig. 3g; p = 0.041), but no differences in uterine-draining lymph nodes were observed between tregitopes and controls (Fig. 3f,h). However, in uterine-draining lymph nodes, lower levels of IL-10 MFI (p = 0.0055) were found in the examined cells in mice treated with tregitope 167 in comparison to the control group (Fig. 3j).

No differences were observed in the frequency of CD4^+^CD25^+^FOXP3^+^ cells (Tregs) on the 14^th^ day of pregnancy in any of the examined tissues (Fig. 3a,b). Nevertheless, T289 administration decreased the percentage of CD4^+^CD25^+^FOXP3^+^ cells in the CD4^+^ splenocyte population (Fig. 3c; p = 0.0003). Analysis of CD4^+^CD25^+^FOXP3^+^IL-10^+^ cells on the 14^th^ day of pregnancy revealed that individual administration of T167 (p = 0.004) and T289 (p = 0.0008) increased the frequency of these cells among splenocytes (Fig. 3e; p = 0.004 or p = 0.0008, respectively) and CD4^+^ cells (Fig. 3g; p = 0.008 or p = 0.0047, respectively). At the same time, administration of tregitopes also significantly enhanced the level of IL-10 (the MFI value) in splenic Tregs (T167 p = 0.0422; T289 p = 0.0155) in comparison to the control (Fig. 3i). We also found that T167 augmented not only the frequency of IL-10-producing Treg cells (Fig. 3f; p = 0.0264) but also the level of IL-10 in uterine-draining lymph nodes on the 14^th^ day of pregnancy (Fig. 3j; p = 0074) in comparison to the vehicle group.

### Tregitopes administration enhanced IL-10 production by Bregs cells

**Figure 4:**
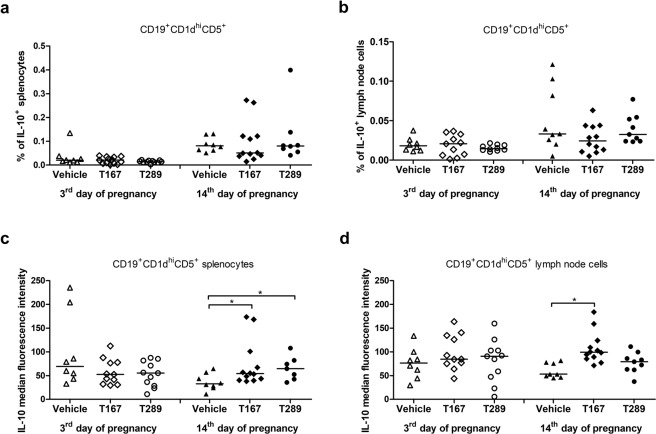
Effect of tregitope treatment on regulatory B lymphocytes in a murine abortion-prone pregnancy model. Cells were stimulated with PMA and ionomycin in the presence of brefeldin A and monensin and the frequencies of CD19^+^CD1d^+^CD5^+^IL-10^+^ (**a)** splenocytes and **(b)** uterine-draining lymph node cells, and the production of IL-10 by CD19^+^CD1d^+^CD5^+^ (**c)** splenocytes and **(d)** lymph node cells were analysed by intracellular staining. The data were analysed by one-way ANOVA (normal distribution) or the Kruskal-Wallis test (non-normal distribution) with Dunn’s multiple comparison post hoc test (P < 0.05) and are presented as individual values with median (at 14dpc n = 12 for T167, n = 9 for T289, n = 9 for Vehicle; at 3dpc n = 11 for T167, n = 11 for T289, n = 9 for Vehicle). *P < 0.05, **P < 0.01, and ***P < 0.001.

To verify whether tregitopes administration stimulates the expansion of B regulatory lymphocyte populations in abortion-prone mice, we examined the percentage of CD19^+^CD5^+^CD1d^hi^IL-10^+^ cells (Bregs) in the spleens and uterine-draining lymph nodes of the treated mice (Fig. 4) based on the gating strategy shown in Fig. 2. No differences in the frequency of Bregs in the spleens (Fig. 4a) or uterine-draining lymph nodes (Fig. 4b) of the treated animals were observed at the 3^rd^ or 14^th^ days of pregnancy in comparison to mice that received PBS (p > 0.05). However, at the 14^th^ day of pregnancy, CD19^+^CD5^+^CD1d^hi^ splenocytes from mice treated with T167 or T289 were found to produce higher levels of IL-10 (Fig. 4c) than CD19^+^CD5^+^CD1d^hi^ splenocytes from control mice (T167 p = 0.0385; T289 p = 0.0462). Moreover, T167 significantly enhanced the level of IL-10 in CD19^+^CD5^+^CD1d^hi^ cells in uterine-draining lymph nodes (Fig. 4d; p = 0.0004).

### Tregitopes administration modulates the costimulatory phenotype of APCs

**Figure 5:**
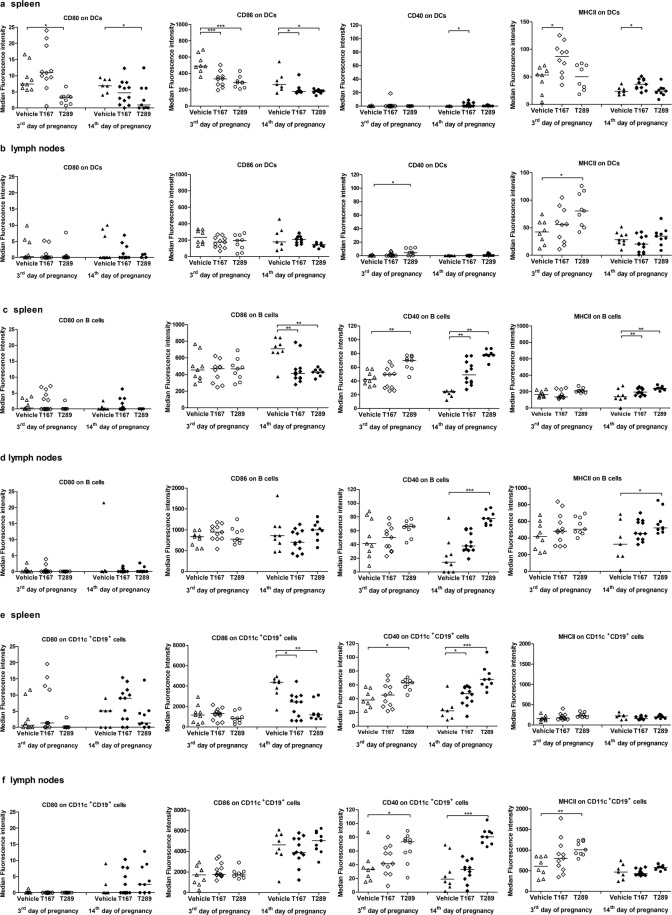
Effect of tregitope treatment on the expression of costimulatory molecules on antigen-presenting cells in abortion-prone mice. The data shown the specific fluorescence intensity of CD40, CD80, CD86 and MHC class II proteins on the surfaces of CD11c^+^ **(a)** splenocytes and **(b)** uterine-draining lymph node cells, CD19^+^ **(c)** splenocytes and **(d)** uterine-draining lymph node cells and CD11c^+^ CD19^+^ **(e)** splenocytes and **(f)** uterine-draining lymph node cells at the 3^rd^ and 14^th^ days of pregnancy. The data were analysed by one-way ANOVA (normal distribution) or the Kruskal-Wallis test (non-normal distribution) with Dunn’s multiple comparison post hoc test (P < 0.05) and are presented as individual values with median (at 14dpc n = 12 for T167, n = 9 for T289, n = 9 for Vehicle; at 3dpc n = 11 for T167, n = 11 for T289, n = 9 for Vehicle). *P < 0.05, **P < 0.01, and ***P < 0.001.

**Figure 6:**
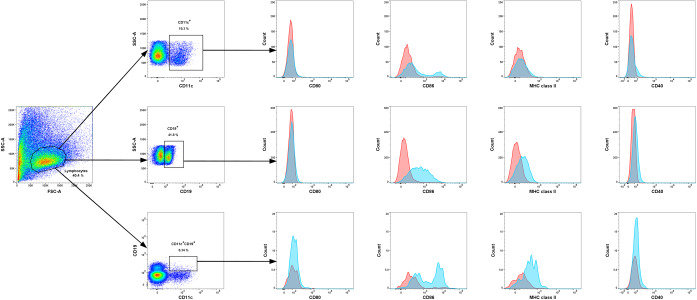
Representative overlay histograms comparing the expression of CD40, CD80, CD86 and MHC class II molecules (blue histograms) with that in the respective isotype-matched controls (red histograms) in CD11c^+^ cells, CD19^+^ cells, and CD19^+^CD11c^+^ cells derived from spleen of abortion-prone mice.

Cousens *et al*. proposed that tregitopes are able to modify the costimulatory phenotype of antigen-presenting cells^22^. To test that hypothesis in our model, we selected dendritic cells (CD11c^+^, DCs), B lymphocytes (CD19^+^) and B cells with expression of CD11c (CD19^+^CD11c^+^) as populations of antigen-presenting cells and examined the surface expression of CD40, CD80, CD86 and MHC class II by measuring the median fluorescence intensity (MFI) of the cells (Fig. 5, Supplementary Table S1) based on the gating strategy shown in Fig. 6. The proportions of antigen-presenting cells with expression of the aforementioned costimulatory molecules are shown in Supplementary Fig. S1.

We found that at the 3^rd^ day of pregnancy, CD11c^+^ splenocytes from mice treated with tregitopes 167 and 289 showed decreased expression of CD86 (p = 0.0004 and p = 0.0001, respectively) compared with CD11c^+^ splenocytes from control mice (Fig. 5a). At the same time, T167 treatment increased MHC class II expression (p = 0.0126), and T289 decreased CD80 expression (p = 0.0178) on splenic CD11c^+^ cells (Fig. 5a). In mice treated with T167, there were no differences in the expression of costimulatory molecules or MHC class II on CD19^+^ (Fig. 5c) and CD19^+^CD11c^+^ (Fig. 5e) splenocytes. In contrast, T289 enhanced the expression of CD40 on CD19^+^ (Fig. 5c; p = 0.0013) and CD19^+^CD11c^+^ splenocytes (Fig. 5e; p = 0.0148). In uterine-draining lymph nodes, we observed no differences in the expression of selected molecules on CD19^+^, CD11c^+^ and CD19^+^CD11c^+^ cells in mice treated with tregitope 167 and controls (Fig. 5b,d,f respectively). However, tregitope 289 increased the expression of MHC class II and CD40 on CD11c^+^ (Fig. 5b; p = 0.017 and p = 0.0195, respectively) and CD19^+^CD11c^+^ cells (Fig. 5f; p = 0.0022 and p = 0.0211, respectively) in uterine-draining lymph nodes.

At the 14^th^ day of pregnancy, mice treated with tregitope 167 showed decreased expression of CD86 (p = 0.014 on CD11c^+^ cells and p = 0.0085 on CD19^+^ cells) and enhanced expression of MHC class II (p = 0.0432 on CD11c^+^ cells and p = 0.0085 on CD19^+^ cells) and CD40 (p = 0.0203 on CD11c^+^ cells and p = 0.0316 on CD19^+^ cells) on splenic CD11c^+^ (Fig. 5a) and CD19^+^ cells (Fig. 5c). T167 also inhibited the expression of CD86 (p = 0.0141) and enhanced CD40 (p = 0.0134) expression on splenic CD19^+^CD11c^+^ cells in comparison with mice treated with vehicle (Fig. 5e). At the same time, tregitope 289 decreased the expression of CD80 (p = 0.013) and CD86 (p = 0.037) on splenic CD11c^+^ cells compared to controls (Fig. 5a). In addition, we observed inhibition of CD86 (p = 0.0483 on CD19^+^ cells and p = 0.0028 on CD19^+^CD11c^+^ cells) and enhancement of CD40 (p < 0.0001) expression on splenic CD19^+^ (Fig. 5c) and CD19^+^CD11c^+^ (Fig. 5e) cells as well as enhancement of MHC class II expression on CD19^+^ cells (Fig. 5c; p = 0.007). In uterine-draining lymph nodes, there was no difference in the expression of costimulatory molecules on selected APCs after T167 administration (Fig. 5b,d,f). In contrast, T289 administration increased the expression of MHC class II (p = 0.0433) and CD40 (p < 0.0001) on B cells (Fig. 5d) and enhanced the expression of CD40 (p < 0.0001) on CD19^+^CD11c^+^ cells (Fig. 5f) in uterine-draining lymph nodes.

## Discussion

It is well known that improper immune tolerance towards foetal antigens can result in miscarriage. IVIg infusion is one of the therapies currently used to increase the live birth rate in women suffering from pregnancy complications/failures. However, the results of IVIg studies are still inconclusive. Despite promising results in some studies, meta-analysis and systematic reviews of clinical trials of IVIg have failed to prove its effectiveness in pregnancy outcome^42–46^. In general, IVIg treatment is relatively safe; however, its high cost and limited supply makes it desirable to develop a new therapy that can be used to improve immune tolerance in pregnancy. The possibility of such a therapy arose in 2008 when the complete amino acid sequence of IgG was determined and natural Treg epitopes, which display high-affinity binding to human class II major histocompatibility complexes (HLA-DR), were discovered^21^. These natural Treg epitopes, called tregitopes, are conserved in all IgG allotypes; they have the ability to bind to various HLAs with high affinity and, like IVIg therapy, are capable of causing Treg expansion.

To investigate the involvement of tregitopes in pregnancy maintenance, we used a commonly studied murine model of immune-mediated pregnancy failure (CBA/J female mice mated with DBA/2 J males) that is believed to share some similarities with human pregnancy loss^47–49^. Similarly to human pregnancies, in this model the key role in suppressing the response towards foreign foetal antigens is played by regulatory T cells that express the Foxp3 transcription factor. In women who suffer from spontaneous miscarriages and preeclampsia, there is a decrease in the number and activity of Treg^28,50,51^. In a murine abortion-prone model, reduced frequencies of Treg cells were observed both at the periphery and locally in the uterus during the pre- and post-implantation period of pregnancy compared with females presenting a normal pregnancy^32,52^.

Using the aforementioned model, we analysed pregnancy outcome at 14 dpc after administration of tregitopes by assessing the numbers of healthy and resorbed foetuses. Our findings showed that administration of either of the tested tregitopes led to a significant reduction in the foetal death rate compared to the control. The unchanged number of viable embryos and decreased number of total implantation sites between groups of mice, may suggest that tregitope’s treatment rather than rescuing the pregnancy, may ensure that only good quality embryos are implanted. Implantation of those “healthy” embryos may be beneficial for mother’s organism to avoid an unnecessary energy investment in developing pregnancy that is doomed to fail^53^. One possible explanation for the effect of tregitopes could be induction of tolerance through increased frequencies of Tregs. We observed that after administration of tregitopes, the peripheral splenic Tregs pool was already higher in early pregnancy (3 dpc), that is, before implantation even occurs, in comparison to the vehicle group. However, only T167 increased the frequency of Tregs producing IL-10 compared to controls. The higher proportion of splenic Tregs expressing IL-10 was maintained in the subsequent stage of pregnancy (14 dpc, mid-pregnancy) after injection of either tregitope in comparison to mice that received vehicle. On the other hand, in paraortal uterine-draining lymph nodes (PALN), only T167 significantly induced Tregs producing IL-10 and also enhanced the production (MFI) of IL-10 compared to control mice. The limited changes in the Tregs proportions in paraortal uterine-draining lymph nodes suggest that tregitopes treatment induce systemic changes in Tregs population, but the specific impact on the IL-10^+^ population in PALN. According to Langenhorst *et al*. Tregs (FOXP3^+^) lymphocytes producing IL-10 are fully activated, terminally differentiated cells with a limited life-span^54^. Therefore, observed by us the presence of this rare phenotype in uterine-draining lymph nodes indicates that the intraperitoneal administration of tregitopes can induce not only peripherally but also locally effective immunosuppression. Thus, it is tempting to speculate that T167 is more effective than T289 in the induction of Tregs. Our data support other studies that have utilized tregitopes 167 and 289 to treat diseases with immune aetiology. In those studies, increased Tregs frequency was also observed after the administration of tregitopes^21,23,24^. Here, we demonstrate that most probably these Tregs are functional and activated as they produce more IL-10 than is found in control mice. The production of IL-10 is one of the main attributes of tolerance induction by Tregs. The importance of the anti-inflammatory cytokine IL-10 in normal pregnancy development has been confirmed in humans as well as in murine studies^55,56^. It was shown that the administration of exogenous IL-10 caused a decrease in pregnancy loss in a murine abortion-prone model^57^; this is one of the mechanisms that may be responsible for the observed decrease in the foetal death rate.

In this paper, we were also interested in whether tregitopes affect another type of regulatory cells, regulatory B lymphocytes (Bregs). The role of B cells in pregnancy has been studied extensively^17,58–60^. The regulatory and suppressive functions of B cells in pregnancy have been mainly attributed to the production of IL-10 and interaction with T cells to inhibit the immune response. In mice, IL-10-producing B cells (B10 cells) were found mainly among CD19^+^CD1d^+^CD5^+^ cells; however, other subsets of B cells may also have suppressive/regulatory functions. In an abortion-prone model, a diminished percentage of splenic CD19^+^IL-10^+^ was observed compared to mice presenting normal pregnancies. Transfer of B10 cells into these mice prevented foetal resorption and led to Tregs expansion^36^. It is believed that the main function of Bregs is control of Tregs function via direct cell-to-cell contact that is mediated by costimulatory molecules and/or indirectly through the cytokine milieu, e.g., the secretion of soluble factors such as IL-10 or TGFβ. Here, we observed that both tregitopes augmented IL-10 expression in CD19^+^CD1d^+^CD5^+^ cells (calculated as MFI) at mid-pregnancy. The results presented here suggest that tregitopes may modulate the cross-talk between Bregs and Tregs and that such cross-talk is important in rescuing foetuses from immune rejection. The tregitopes-induced elevated expression of IL-10 in both Tregs and Bregs suggests that indirect contact between these cells may be involved. However, their interplay is probably more complex, as we also found changes in the expression of costimulatory molecules on B cells after the administration of tregitopes.

At present, it is undebatable that in addition to producing autoantibodies and pro- and anti-inflammatory cytokines, B cells, along with dendritic cells (DCs), are professional antigen- presenting cells that are able to present antigens to T cells in the context of MHC class II molecules. The antigen presentation, together with high expression of MHCII, costimulatory molecules (CD80, CD86) and secretion of cytokines, leads to T cell proliferation, cytokine production and the development of effector functions. Antigen presentation by APCs in the absence of a costimulatory signal may cause T cells to differentiate into Tregs. Here, we found that at the preimplantation stage of pregnancy both tregitopes caused decreased expression of CD86 and CD80 on CD11c^+^ cells. It is well known that low expression of maturation markers as well as costimulatory molecules is a determinant of the immature state of DCs (iDCs) that enables the establishment of tolerance toward self-antigens^61,62^. Therefore, we hypothesize that these tregitopes may trigger an immature status of DCs and that these DCs in turn effectively induce Treg expansion upon antigen presentation. The iDCs phenotype is maintained even at mid-pregnancy, as we observed decreased expression of CD86 on CD11c^+^ cells and other APCs (CD19^+^). On the other hand, we observed the enhancement of CD40 and MHC class II molecules on splenic and lymph node APCs during the course of pregnancy after the administration of tregitopes. This may suggest that DCs became activated rather than being immature and indicate the process of activation of T cells. However, it was shown that increased expression of MHC II molecule on DCs^63,64^ enable T cell activation that may contribute to Tregs development as its enhancement is required for Treg homeostasis^65^. Furthermore, the immature/mature form of DCs depend not only on costimulatory molecules but also on timing, dose and signal strength of many factors that interact with DCs like the inflammatory cytokines or chemokines^66^. Therefore, we believe that the observed increased production of IL-10 by Bregs and Tregs may lead to DCs shift towards iDCs because it was previously shown that IL-10 produced by B10 cells is responsible for keeping DCs in an immature state and that it inhibits their ability to present antigens to T cells^67^. However, further studies, including detailed DCs phenotyping, needs to be performed to clarify the DCs state after tregitopes administration. Like IL-10, TGFβ is one of the cytokines that is crucial in controlling the phenotype of DCs and the differentiation of Tregs^68–70^. We found that the concentration of plasma TGFβ1 during early pregnancy is higher in mice treated with T289. We believe that TGFβ may be another factor that is involved in the induction and maintenance of Tregs and that it may be beneficial for pregnancy maintenance after administration of tregitopes.

The downregulation of CD80 and CD86 observed in this study may partially explain the findings of Jin *et al*. and Zhou *et al*. that systemic blockade of CD80 and CD86 or of CD86 alone at implantation inhibits maternal rejection of allogenic foetuses in CBA/JxDBA/2 J matings^71,72^. We hypothesize that the alteration in the expression of CD86 and CD80 on APCs, including splenic B cells, that is observed after the administration of tregitopes may be crucial for proper embryo implantation and subsequent embryonic development through activation of immune tolerance toward foetal antigens. Our results confirmed data obtained in previous studies that also demonstrated downregulation of CD80 and CD86 costimulatory molecules on APCs after exposure to tregitopes^22^. All of these findings support the hypothesis proposed by Cousens *et al*. that tregitopes may generate immune tolerance to self-antigens and probably to alloantigens through their presentation by APCs.

## Conclusions

In summary, our study demonstrated that treatment with tregitopes significantly increased the frequency of Tregs, enhanced the production of IL-10 by Tregs and Bregs and changed the costimulatory responses of APCs, contributing to the reduction of the foetal death rate. Our findings for the first time indicate, that tregitopes may be a potential tool for therapeutic intervention in cases of immune-mediated pregnancy failures. However, further studies should be performed to confirm the ability of tregitopes to modulate the immune response in humans, especially in the context of IVIg therapies.

## Methods

### Animals

CBA/J female and DBA/2 J male mice (Charles River Laboratories, Sulzfeld, Germany), were housed under specific pathogen-free (SPF) conditions under/in a dark-light cycle of 12 h:12 h. The reproductive cycle of 6-8-week-old female CBA/J mice was monitored by Cytocolor staining (Merck Millipore, USA) of vaginal smears. Females at the proestrus phase were placed with stud DBA/2 J males at 19:00 and copulatory plug were checked at 7:00-8:00. The morning the plug appears was defined as day 0 *post coitum* (dpc). Two periods of pregnancy were investigated: 3^rd^ day of pregnancy (preimplantation pregnancy) and 14^th^ day of pregnancy. Three groups of mice for each period of pregnancy were examined: a group of pregnant mice after administration of tregitope 167, a group of pregnant mice after administration tregitope 289 or a group of pregnant mice after administration PBS buffer. Immediately after detection of a copulatory plug, female mice were intraperitoneally injected with 100 µg (dissolved in 150 µl PBS) of mouse tregitope 167 (T167) with a sequence corresponding to the Fc fragment of IgG (PAVLQSDLYTLSSSVTVPSS, purity >90%, GeneCust, Luxembourg), 100 µg of mouse tregitope 289 (T289) with a sequence corresponding to Fc fragment of IgG (EEQFNSTFRSVSELPIMHQ, purity >90%, GeneCust, Luxembourg) or phosphate-buffered saline (vehicle) as a control. The tregitopes concentrations were selected based on our previous observations^73^ and other studies that used tregitopes to treat autoimmune diseases in mouse models^23–25^. Pregnancy was confirmed *post mortem* by the presence of embryos in the uterine horns after flushing at 3 dpc or by observation of the number of foetuses and implantation sites at 14 dpc. At the 3^rd^ and 14^th^ days of pregnancy, the pregnant mice were anaesthetized, blood samples were collected, and the animals were euthanized by cervical dislocation. The spleens, the paraortal uterine-draining lymph nodes (PALN) and the uteri were dissected for further analyses. All efforts were made to minimize the animals’ suffering. The animal experiments were approved by the Local Ethics Committee for Experiments on Animals at the Hirszfeld Institute of Immunology and Experimental Therapy in Wroclaw (No. 53/2015). All methods used in this study were performed in accordance with the relevant guidelines and regulations.

### Tissue processing

The animals’ spleens and lymph nodes were processed according to a standard protocol as described previously^73,74^. In brief, spleens and uterine-draining lymph nodes were squeezed through a 40-μm cell strainer (Falcon) into 0.84% ammonium chloride solution and sorting buffer (PBS buffer supplemented with 2 mM EDTA and 2% foetal bovine serum (Biowest, France)), respectively, and washed twice (4 °C, 300×g, 10 minutes) in sorting buffer. The isolated cells were used in flow cytometry analysis.

Uteri from 14 dpc pregnant females were used to determine the foetal death rate. The rate was calculated as the number of resorbed embryos divided by the total number of embryos (resorbed plus viable embryos) multiplied by one hundred. The abortion sites were identified by their small size accompanied by a necrotic, haemorrhagic appearance compared with normal embryos and placentas^32^.

### Flow cytometry

Splenic and lymph node cells (1×10^6^ cells) were stimulated with 0.1 µg/ml phorbol 12-myristate 13-acetate (Cayman Chemical, USA), 1 µg/ml ionomycin (Cayman Chemical, USA), 10 µg/ml brefeldin A (eBioscience, USA) and 2 µM monensin (eBioscience, USA) in RPMI-1640 medium supplemented with 10% FBS and 1x penicillin/streptomycin (from 100×) (Merck Millipore, USA) in a tissue culture incubator at 37 °C and 5% CO_2_ for 6 hours. After incubation, the cells were stained as previously described^75^. In brief, the cells were stained with anti-mouse CD4 Alexa Fluor 700 (eBioscience, USA, clone: Gk1.5), CD25 APC-Cy7 (BD Biosciences, clone: PC61), CD19 FITC (eBioscience, USA, clone: eBio1D3), CD1d PE (eBioscience, USA, clone: 1B1), CD5 Pacific Blue (eBioscience, USA, clone: 53-7.3), CD11c APC-Cy7(eBioscience, USA, clone: N418), CD80 Pacific Blue (eBioscience, USA, clone: 16-10A1), CD86 PE-Cy7 (eBioscience, USA, clone: GL1), CD40 PE (eBioscience, USA, clone: 1C10) and MHC Class II (IA/IE) Alexa Fluor 700 (eBioscience, USA, clone: M5/114.15.2) antibodies or appropriate isotype controls at 4 °C for 30 minutes in the dark. The cells were then washed twice with staining buffer, and intracellular staining was performed according to the instructions supplied with the FOXP3/Transcription Factor Staining Buffer Set (eBioscience, USA). In brief, the cells were fixed for 14 h at 4 °C in the dark, washed twice with permeabilization buffer (eBioscience, USA) and stained for 15 minutes at 4 °C with anti-CD16/CD32 antibodies to block nonspecific binding. The cells were then stained for 1 h at 4 °C in the dark with anti-mouse FOXP3 PE-Cy7(eBioscience, USA, clone: FjK-16s) and anti-IL-10 APC (eBioscience, USA, clone: JES5-16E3) antibodies or the appropriate isotype control at the same concentration as the specific antibody. The cells were washed twice with permeabilization buffer, and cellular fluorescence was immediately measured on an LSRFortessa cell analyzer (Becton Dickinson, USA). The relative levels of CD40, CD80, CD86, MHC-class II and IL-10 antigens are shown as the specific median fluorescence intensity (MFI) based on the difference between the median fluorescence intensity of the specifically stained cells and the isotype-matched control cells gated for the populations of interest. The MFI of CD40, CD80, CD86, MHC-class II molecules were measured/calculated in total: B cells, DCs (CD11c^+^ cells) and CD11c^+^CD19^+^ cells. The MFI of IL-10 were measured/calculated in CD4^+^CD25^+^FOXP3^+^cells and CD19^+^CD1d^+^CD5^+^ cells. All analyses were conducted using Weasel 3.0.2 software (Walter and Eliza Hall Institute, Parkville, Australia).

### Cytokines ELISA

Blood samples were collected from all examined mice and centrifuged at 10,000 × g at 4 °C for 10 minutes. The obtained sera were stored at −80 °C. The concentrations of TGFβ1, IFN-γ, IL-2, IL-4 and IL-10 in the sera were measured using the murine Ready-SET-Go! kit (eBioscience, USA) according to the manufacturer’s instructions. Briefly, 96-well ELISA plates were coated with specific antibodies overnight at 4 °C and then blocked for 1 hour at room temperature (RT). Sera diluted 1:2 (IFN-γ, IL-2, IL-4 and IL-10) or 1:5 (TGFβ1) and standard concentrations of cytokines (100 μl/well) were then added to the wells, and the plates were incubated overnight at 4 °C. The wells were then washed three times and incubated with biotinylated specific antibodies for 60 minutes at RT. After further triple washing, horseradish peroxidase-conjugated streptavidin was added, and the plates were incubated for 30 minutes at RT. The washed plates were incubated for 10 minutes in the dark at RT with TMB substrate, and the reaction was stopped with 50 μl of 1 M H_2_SO_4_. The absorbance (A_450_) was measured on a Wallac 1420 Victor2 Microplate Reader (PerkinElmer, USA) within 15 minutes of the endpoint of the protocol.

### Statistical analysis

Statistical calculations were performed in GraphPad Prism 7 (GraphPad Software, USA). Data distribution was assessed using the Shapiro-Wilk normality test. Homoscedasticity was tested with Brown-Forsythe test. One-way ANOVA (parametric) or the Kruskal-Wallis test (nonparametric) with Dunn’s multiple comparison post hoc test were performed according to the data distribution. A p value <0.05 was considered statistically significant.

## Supplementary information

Supplementary information.

Supplementary information 2.

## Data Availability

The datasets generated during and/or analysed during the current study are available from the corresponding author on reasonable request.

## References

[1] Evaluation and treatment of recurrent pregnancy loss: a committee opinion. *Fertil. Steril*. **98**, 1103–1111 (2012). 10.1016/j.fertnstert.2012.06.048 22835448

[2] Larsen EC, Christiansen OB, Kolte AM, Macklon N (2013). New insights into mechanisms behind miscarriage. BMC Med..

[3] Saito S, Nakashima A, Shima T (2011). Future directions of studies for recurrent miscarriage associated with immune etiologies. J. Reprod. Immunol..

[4] RPL, T. E. G. G. on *et al*. ESHRE guideline: recurrent pregnancy loss. Hum. Reprod. Open 2018, hoy004 (2018). 10.1093/hropen/hoy004 PMC6276652 31486805

[5] Coulam CB (1994). Immunotherapy with intravenous immunoglobulin for treatment of recurrent pregnancy loss: American experience. Am. J. Reprod. Immunol..

[6] Coulam CB, Krysa L, Stern JJ, Bustillo M (1995). Intravenous immunoglobulin for treatment of recurrent pregnancy loss. Am. J. Reprod. Immunol.

[7] Mueller-Eckhardt G (1994). Immunotherapy with intravenous immunoglobulin for prevention of recurrent pregnancy loss: European experience. Am. J. Reprod. Immunol..

[8] Yamada H (2012). A high dose intravenous immunoglobulin therapy for women with four or more recurrent spontaneous abortions. ISRN Obstet. Gynecol..

[9] Vaquero E (2001). Pregnancy outcome in recurrent spontaneous abortion associated with antiphospholipid antibodies: a comparative study of intravenous immunoglobulin versus prednisone plus low-dose aspirin. Am. J. Reprod. Immunol..

[10] Bozic Antic I (2014). Recurrent spontaneous abortions, Hashimoto thyroiditis and alopecia totalis: response to anticoagulation and intravenous immunoglobulin therapy. Gynecol. Endocrinol..

[11] Christiansen OB (1995). Placebo-controlled trial of treatment of unexplained secondary recurrent spontaneous abortions and recurrent late spontaneous abortions with i.v. immunoglobulin. Hum. Reprod.

[12] Jauniaux E, Farquharson RG, Christiansen OB, Exalto N (2006). Evidence-based guidelines for the investigation and medical treatment of recurrent miscarriage. Hum. Reprod..

[13] Stephenson MD, Dreher K, Houlihan E, Wu V (1998). Prevention of unexplained recurrent spontaneous abortion using intravenous immunoglobulin: a prospective, randomized, double-blinded, placebo-controlled trial. Am. J. Reprod. Immunol.

[14] Nyborg KM, Kolte AM, Larsen EC, Christiansen OB (2014). Immunomodulatory treatment with intravenous immunoglobulin and prednisone in patients with recurrent miscarriage and implantation failure after *in vitro* fertilization/intracytoplasmic sperm injection. Fertil. Steril..

[15] Schwab I, Nimmerjahn F (2013). Intravenous immunoglobulin therapy: how does IgG modulate the immune system?. Nat. Rev. Immunol..

[16] Baerenwaldt A, Biburger M, Nimmerjahn F (2010). Mechanisms of action of intravenous immunoglobulins. Expert Rev. Clin. Immunol..

[17] Fettke F, Schumacher A, Costa S-D, Zenclussen AC (2014). B cells: the old new players in reproductive immunology. Front. Immunol.

[18] Han AR, Lee SK (2018). Immune modulation of i.v. immunoglobulin in women with reproductive failure. Reprod. Med. Biol.

[19] Padet L, Bazin R (2013). IVIg prevents the in vitro activation of T cells by neutralizing the T cell activators. Immunol. Lett..

[20] Kessel A (2007). Intravenous immunoglobulin therapy affects T regulatory cells by increasing their suppressive function. J. Immunol..

[21] De Groot AS (2008). Activation of natural regulatory T cells by IgG Fc-derived peptide ‘Tregitopes’. Blood.

[22] Cousens L, Najafian N, Martin WD, De Groot AS (2014). Tregitope: Immunomodulation powerhouse. Hum. Immunol..

[23] Cousens LP (2013). Application of IgG-derived natural Treg epitopes (IgG Tregitopes) to antigen-specific tolerance induction in a murine model of type 1 diabetes. J. Diabetes Res..

[24] Elyaman W, Khoury SJ, Scott DW, De Groot AS (2011). Potential application of tregitopes as immunomodulating agents in multiple sclerosis. Neurol. Res. Int.

[25] Prangtaworn P (2018). Tregitope-linked Refined Allergen Vaccines for Immunotherapy in Cockroach Allergy. Sci. Rep.

[26] Saito S, Sasaki Y, Sakai M (2005). CD4(+)CD25high regulatory T cells in human pregnancy. J. Reprod. Immunol..

[27] Aluvihare VR, Kallikourdis M, Betz AG (2004). Regulatory T cells mediate maternal tolerance to the fetus. Nat. Immunol..

[28] Sasaki Y (2004). Decidual and peripheral blood CD4+CD25+ regulatory T cells in early pregnancy subjects and spontaneous abortion cases. Mol. Hum. Reprod..

[29] Schumacher A, Zenclussen AC (2014). Regulatory T cells: regulators of life. Am. J. Reprod. Immunol..

[30] Arruvito L, Sotelo AI, Billordo A, Fainboim L (2010). A physiological role for inducible FOXP3(+) Treg cells. Lessons from women with reproductive failure. Clin. Immunol..

[31] Somerset DA, Zheng Y, Kilby MD, Sansom DM, Drayson MT (2004). Normal human pregnancy is associated with an elevation in the immune suppressive CD25+ CD4+ regulatory T-cell subset. Immunology.

[32] Thuere C (2007). Kinetics of regulatory T cells during murine pregnancy. Am. J. Reprod. Immunol..

[33] Jensen F (2012). CD19+CD5+ cells as indicators of preeclampsia. Hypertens. (Dallas, Tex. 1979).

[34] Rolle L (2013). Cutting edge: IL-10-producing regulatory B cells in early human pregnancy. *Am*. J. Reprod. Immunol..

[35] Muzzio DO, Ziegler KB, Ehrhardt J, Zygmunt M, Jensen F (2016). Marginal zone B cells emerge as a critical component of pregnancy well-being. Reproduction.

[36] Jensen F, Muzzio D, Soldati R, Fest S, Zenclussen AC (2013). Regulatory B10 cells restore pregnancy tolerance in a mouse model. Biol. Reprod.

[37] Wafula PO (2009). PD-1 but not CTLA-4 blockage abrogates the protective effect of regulatory T cells in a pregnancy murine model. Am. J. Reprod. Immunol..

[38] Heine O, Mueller-Eckhardt G, Stitz L, Pabst W (1992). Influence of treatment with mouse immunoglobulin on the rate of viable neonates in the CBA/J x DBA/2 J model. Res. Exp. Med. (Berl).

[39] Takeda M (2007). Administration of high-dose intact immunoglobulin has an anti-resorption effect in a mouse model of reproductive failure. Mol. Hum. Reprod..

[40] Clark DA (2013). Seminal plasma peptides may determine maternal immune response that alters success or failure of pregnancy in the abortion-prone CBAxDBA/2 model. J. Reprod. Immunol..

[41] De Groot AS (2019). Therapeutic administration of Tregitope-Human Albumin Fusion with Insulin Peptides to promote Antigen-Specific Adaptive Tolerance Induction. Sci. Rep.

[42] Christiansen OB (2015). Intravenous immunoglobulin treatment for secondary recurrent miscarriage: a randomised, double-blind, placebo-controlled trial. BJOG.

[43] Hutton B (2007). Use of intravenous immunoglobulin for treatment of recurrent miscarriage: a systematic review. *BJOG An Int*. J. Obstet. Gynaecol..

[44] Wang S-W (2016). The effect of intravenous immunoglobulin passive immunotherapy on unexplained recurrent spontaneous abortion: a meta-analysis. Reprod. Biomed. Online.

[45] Stephenson MD (2010). Intravenous immunoglobulin and idiopathic secondary recurrent miscarriage: a multicentered randomized placebo-controlled trial. Hum. Reprod..

[46] Ata B, Tan SL, Shehata F, Holzer H, Buckett W (2011). A systematic review of intravenous immunoglobulin for treatment of unexplained recurrent miscarriage. Fertil. Steril..

[47] Girardi G, Yarilin D, Thurman JM, Holers VM, Salmon JE (2006). Complement activation induces dysregulation of angiogenic factors and causes fetal rejection and growth restriction. J. Exp. Med.

[48] Ahmed A, Singh J, Khan Y, Seshan SV, Girardi G (2010). A new mouse model to explore therapies for preeclampsia. PLoS One.

[49] Clark DA, Chaouat G, Arck PC, Mittruecker HW, Levy GA (1998). Cytokine-dependent abortion in CBA x DBA/2 mice is mediated by the procoagulant fgl2 prothrombinase [correction of prothombinase]. J. Immunol..

[50] Rahimzadeh M, Norouzian M, Arabpour F, Naderi N (2016). Regulatory T-cells and preeclampsia: an overview of literature. Expert Rev. Clin. Immunol..

[51] Sasaki Y (2007). Proportion of peripheral blood and decidual CD4(+) CD25(bright) regulatory T cells in pre-eclampsia. Clin. Exp. Immunol.

[52] Zenclussen AC (2005). Abnormal T-cell reactivity against paternal antigens in spontaneous abortion: adoptive transfer of pregnancy-induced CD4+CD25+ T regulatory cells prevents fetal rejection in a murine abortion model. Am. J. Pathol..

[53] Teklenburg G, Salker M, Heijnen C, Macklon NS, Brosens JJ (2010). The molecular basis of recurrent pregnancy loss: impaired natural embryo selection. Mol. Hum. Reprod..

[54] Langenhorst D (2012). Sequential induction of effector function, tissue migration and cell death during polyclonal activation of mouse regulatory T-cells. PLoS One.

[55] Robertson SA, Care AS, Skinner RJ (2007). Interleukin 10 regulates inflammatory cytokine synthesis to protect against lipopolysaccharide-induced abortion and fetal growth restriction in mice. Biol. Reprod.

[56] Wilson R (2004). Abnormal cytokine levels in non-pregnant women with a history of recurrent miscarriage. Eur. J. Obstet. Gynecol. Reprod. Biol..

[57] Chaouat G (1995). IL-10 prevents naturally occurring fetal loss in the CBA x DBA/2 mating combination, and local defect in IL-10 production in this abortion-prone combination is corrected by *in vivo* injection of IFN-tau. J. Immunol..

[58] Guzman-Genuino RM, Diener KR (2017). Regulatory B Cells in Pregnancy: Lessons from Autoimmunity, Graft Tolerance, and Cancer. Front. Immunol.

[59] Nguyen TG, Ward CM, Morris JM (2013). To B or not to B cells-mediate a healthy start to life. Clin. Exp. Immunol..

[60] Esteve-Sole, A. *et al*. B Regulatory Cells: Players in Pregnancy and Early Life. *Int*. *J. Mol. Sci*. **19** (2018). 10.3390/ijms19072099 PMC6073150 30029515

[61] Morva A (2012). Maturation and function of human dendritic cells are regulated by B lymphocytes. Blood.

[62] Dudek AM, Martin S, Garg AD, Agostinis P (2013). Immature, Semi-Mature, and Fully Mature Dendritic Cells: Toward a DC-Cancer Cells Interface That Augments Anticancer Immunity. Front. Immunol.

[63] LeibundGut-Landmann S, Waldburger J-M (2004). Reis e Sousa, C., Acha-Orbea, H. & Reith, W. MHC class II expression is differentially regulated in plasmacytoid and conventional dendritic cells. Nat. Immunol..

[64] LeibundGut-Landmann S (2004). Mini-review: Specificity and expression of CIITA, the master regulator of MHC class II genes. Eur. J. Immunol..

[65] Irla M (2010). MHC class II-restricted antigen presentation by plasmacytoid dendritic cells inhibits T cell-mediated autoimmunity. J. Exp. Med.

[66] Audiger C, Rahman MJ, Yun TJ, Tarbell KV, Lesage S (2017). The Importance of Dendritic Cells in Maintaining Immune Tolerance. J. Immunol..

[67] Matsushita T, Horikawa M, Iwata Y, Tedder TF (2010). Regulatory B cells (B10 cells) and regulatory T cells have independent roles in controlling experimental autoimmune encephalomyelitis initiation and late-phase immunopathogenesis. J. Immunol..

[68] Fantini MC (2004). Cutting edge: TGF-beta induces a regulatory phenotype in CD4+CD25- T cells through Foxp3 induction and down-regulation of Smad7. J. Immunol..

[69] Chen W (2003). Conversion of peripheral CD4+CD25- naive T cells to CD4+CD25+ regulatory T cells by TGF-beta induction of transcription factor Foxp3. J. Exp. Med..

[70] Ghaebi M (2017). Immune regulatory network in successful pregnancy and reproductive failures. Biomed. Pharmacother..

[71] Jin L-P, Zhou Y-H, Wang M-Y, Zhu X-Y, Li D-J (2005). Blockade of CD80 and CD86 at the time of implantation inhibits maternal rejection to the allogeneic fetus in abortion-prone matings. J. Reprod. Immunol..

[72] Zhu X-Y (2005). Blockade of CD86 signaling facilitates a Th2 bias at the maternal-fetal interface and expands peripheral CD4+CD25+ regulatory T cells to rescue abortion-prone fetuses. Biol. Reprod..

[73] Okoniewska KM (2017). New tregitopes inducing adaptive regulatory T cells in mice. J. Physiol. Pharmacol..

[74] Slawek A, Maj T, Chelmonska-Soyta A (2013). CD40, CD80, and CD86 costimulatory molecules are differentially expressed on murine splenic antigen-presenting cells during the pre-implantation period of pregnancy, and they modulate regulatory T-cell abundance, peripheral cytokine response, and pregnancy outcome. Am. J. Reprod. Immunol..

[75] Lorek D, Kedzierska AE, Slawek A, Chelmonska-Soyta A (2019). Expression of Toll-like receptors and costimulatory molecules in splenic B cells in a normal and abortion-prone murine pregnancy model. *Am*. J. Reprod. Immunol..

