# Supplementary material for: Tregitopes regulate the tolerogenic immune response and decrease the foetal death rate in abortion-prone mouse matings
Source: Sci Rep. 2020 Jun 29;10:10531. doi: 10.1038/s41598-020-66957-z (PMC7324366; doi:10.1038/s41598-020-66957-z)
Supplement: Supplementary file 1 — Supplementary information. [file 41598_2020_66957_MOESM1_ESM.pdf]

# Tregitopes regulate the tolerogenic immune response and decrease the foetal death rate in abortion-prone mouse matings

Anna Ewa Kedzierska, Daria Lorek, Anna Slawek, Anna Chelmonska-Soyta

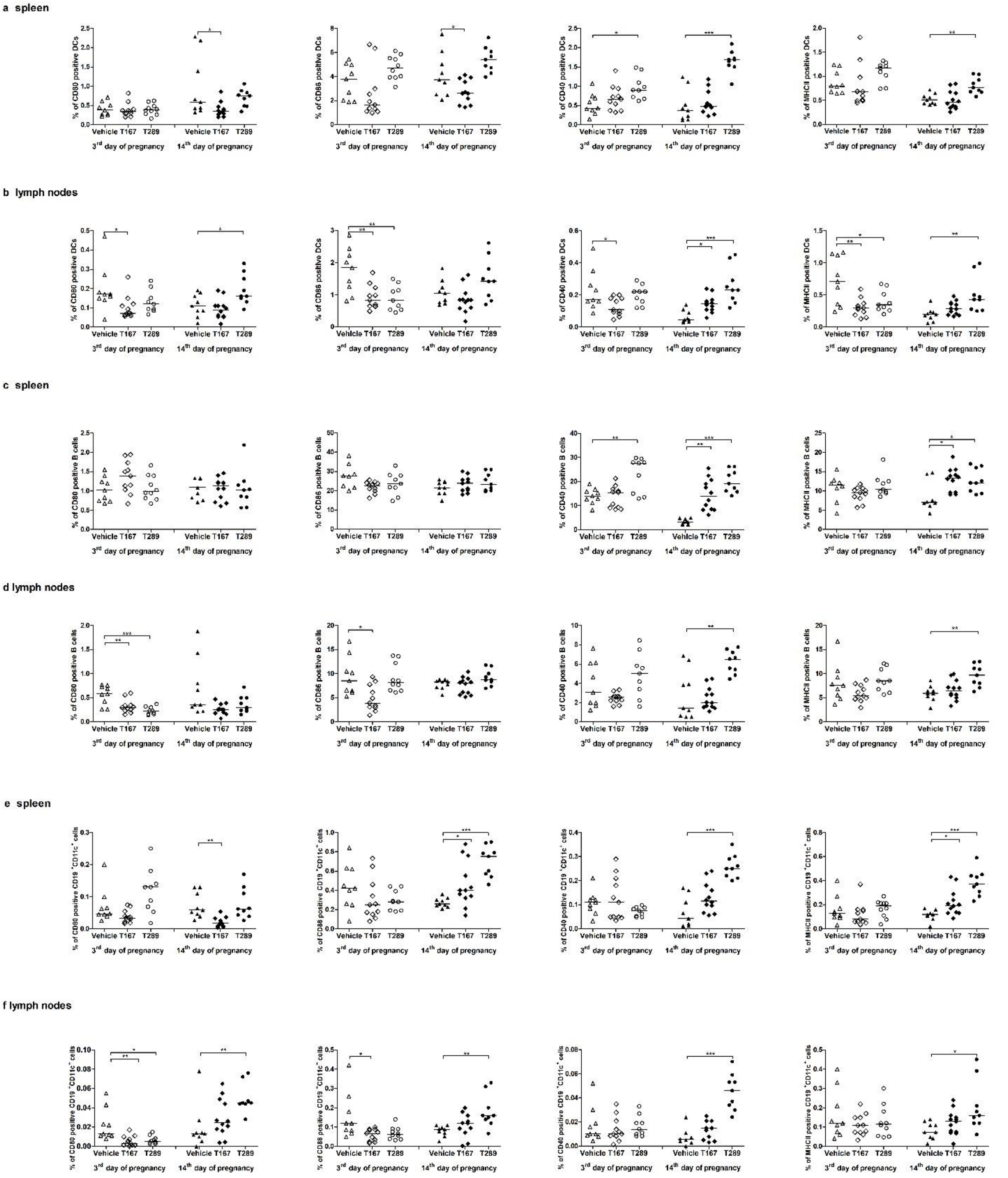


**Supplementary Figure S1.** Effect of tregitope treatment on the antigen-presenting cells with expression of costimulatory molecules in abortion-prone mice. Cells were stimulated with PMA and ionomycin in the presence of brefeldin A and monensin and the frequencies of CD40, CD80, CD86 and MHC class II proteins on the surfaces of CD11c$^{+}$ **(a)** splenocytes and **(b)** uterine-draining lymph

node cells, $CD19^+$ **(c)** splenocytes and **(d)** uterine-draining lymph node cells and $CD11c^+$ $CD19^+$ **(e)** splenocytes and **(f)** uterine-draining lymph node cells at the 3$^{rd}$ and 14$^{th}$ days of pregnancy. The data were analysed by one-way ANOVA (normal distribution) or the Kruskal-Wallis test (non-normal distribution) with Dunn's multiple comparison post hoc test ($P <0.05$) and are presented as individual values with median (at 14dpc n=12 for T167, n=9 for T289, n=9 for Vehicle; at 3dpc n=11 for T167, n=11 for T289, n=9 for Vehicle). *$P < 0.05$, **$P < 0.01$, and ***$P < 0.001$.
