# Supplementary material for: Tregitopes regulate the tolerogenic immune response and decrease the foetal death rate in abortion-prone mouse matings
Source: Sci Rep. 2020 Jun 29;10:10531. doi: 10.1038/s41598-020-66957-z (PMC7324366; doi:10.1038/s41598-020-66957-z)
Supplement: Supplementary file 2 — Supplementary information 2. [file 41598_2020_66957_MOESM2_ESM.pdf]

Tregitopes regulate the tolerogenic immune response and decrease the foetal death rate in abortion-prone mouse matings

Anna Ewa Kedzierska, Daria Lorek, Anna Slawek, Anna Chelmonska-Soyta

	CD11c$^+$				CD19$^+$				CD19$^+$CD11c$^+$			
	**CD80**	**CD86**	**MHCII**	**CD40**	**CD80**	**CD86**	**MHCII**	**CD40**	**CD80**	**CD86**	**MHCII**	**CD40**
**TREGITOPE 167**												
3 dpc spleen		*** ↓	* ↑									
3 dpc LN												
14 dpc spleen		* ↓	* ↑			** ↓	** ↑	** ↑		* ↓		* ↑
14 dpc LN												
**TREGITOPE 289**												
3 dpc spleen	* ↓	*** ↓						** ↑				* ↑
3 dpc LN			* ↑	* ↑							** ↑	* ↑
14 dpc spleen	* ↓	* ↓				* ↓	** ↑	*** ↑		** ↓		*** ↑
14 dpc LN							** ↑	*** ↑				*** ↑

Supplementary Table S1. Summary of expression of costimulatory molecules on antigen-presenting cells in abortion-prone mice. The data show the changes in the specific fluorescence intensity due to CD40, CD80, CD86 and MHC class II proteins on the surfaces of splenic and uterine-draining lymph node cells at the 3rd and 14th days of pregnancy compared to cells obtained from control mice. The data were analysed by one-way ANOVA (normal distribution) or the Kruskal-Wallis test (non-normal distribution) with Dunn's multiple comparison post hoc test ($P < 0.05$). *$P < 0.05$, **$P < 0.01$, and ***$P < 0.001$. Legend: LN: lymph nodes, ↑: increased MFI compared to control mice, ↓: decreased MFI compared to control mice
